# Flaxseed dietary fibers lower cholesterol and increase fecal fat excretion, but magnitude of effect depend on food type

**DOI:** 10.1186/1743-7075-9-8

**Published:** 2012-02-03

**Authors:** Mette Kristensen, Morten G Jensen, Julie Aarestrup, Kristina EN Petersen, Lise Søndergaard, Mette S Mikkelsen, Arne Astrup

**Affiliations:** 1 Department of Human Nutrition, Faculty of Life Sciences, University of Copenhagen, Denmark; 2 Quality & Technology, Department of Food Science, Faculty of Life Sciences, University of Copenhagen, Denmark

**Keywords:** Flaxseed, dietary fiber, fat excretion, cholesterol

## Abstract

**Background:**

Dietary fibers have been proposed to play a role in cardiovascular risk as well as body weight management. Flaxseeds are a good source of dietary fibers, and a large proportion of these are water-soluble viscous fibers.

**Method:**

Here, we examine the effect of flaxseed dietary fibers in different food matrices on blood lipids and fecal excretion of fat and energy in a double-blind randomized crossover study with 17 subjects. Three different 7-d diets were tested: a low-fiber control diet (Control), a diet with flaxseed fiber drink (3/day) (Flax drink), and a diet with flaxseed fiber bread (3/day) (Flax bread). Total fat and energy excretion was measured in feces, blood samples were collected before and after each period, and appetite sensation registered 3 times daily before main meals.

**Results:**

Compared to control, Flax drink lowered fasting total-cholesterol and LDL-cholesterol by 12 and 15%, respectively, (p < 0.01), whereas Flax bread only produced a reduction of 7 and 9%, respectively (p < 0.05). Fecal fat and energy excretion increased by 50 and 23% with Flax drink consumption compared to control (p < 0.05), but only fecal fat excretion was increased with Flax bread compared to control (p < 0.05).

**Conclusion:**

Both Flax drink and Flax bread resulted in decreased plasma total and LDL-cholesterol and increased fat excretion, but the food matrix and/or processing may be of importance. Viscous flaxseed dietary fibers may be a useful tool for lowering blood cholesterol and potentially play a role in energy balance.

**Trial Registration:**

ClinicalTrials.gov: NCT00953004

## Introduction

Prospective cohort studies suggest that consumption of dietary fibers protects against coronary heart disease [1], although all mechanisms are not fully elucidated. The cholesterol lowering effect of soluble viscous dietary fiber, particularly β-glucans from oats and barley has been known for decades [2]. A large number of studies have demonstrated that oat products lower total and LDL cholesterol [3-6]. Likely, this is linked to their ability to increase intraluminal viscosity thereby affecting the entero-hepatic recirculation of bile acids and lipid metabolism[7]. Recently, extracted flaxseed fiber added to bread was found to lower cholesterol in diabetics [8]. Flaxseeds contain ~30% dietary fibers of which one third are water-soluble and belonging to a group of heterogeneoues polysaccharides [9]. Warrand and colleagues [10,11] found that the water extractable neutral monosaccharides from flaxseed were a mixture of three major families of polymers: arabinoxylans with a A/X ratio of ~0.25, and various amount of galactose and fucose residues. Thus, the lower A/X ratio compared to wheat arabinoxylans, which are mainly insoluble, results in different physicochemical properties. Also, flaxseeds contain some pectins. Flaxseed fibers form highly viscous solutions upon hydration, which is similar to those observed for other gums [12,13].

Dietary fibers may also play a role in body weight regulation, through both hunger suppression and diminished nutrient absorption [14]. Alzueta and colleagues (2003) [15] found the flaxseed mucilage to be responsible for diminished growth of broiler chickens when comparing feeds with whole and demucilaged flaxseeds. Similarly, we found that addition of flaxseeds to rye breads (6 g/100 g) significantly reduced the digestibility of fat and energy in humans [16]. Also, extracted flaxseed fibers was found to reduce weight gain and fat digestibility when fed to growing rats in higher doses (10% w/w) (Kristensen M, Knudsen KEB, Jørgensen H, Oomah D, Bügel S, Toubro S, Tetens I, Astrup A. Flaxseed dietary fibers reduce apparent energy and fat digestibility and weight gain in growing rats, submitted). Particularly viscous fibers appear effective in suppression of hunger [17]. They can affect multiple aspects of the gastrointestinal function such as gastric emptying rate and nutrient absorption rate in the small intestine [18], which offers numerous opportunities to influence satiation and satiety. In concordance with this, we observed that doses of 5 and 10 g of flaxseed fibers increased satiety and gave a prolonged decrease in ghrelin, a hunger-signaling gut peptide [19].

In the present study we test the hypothesis that addition of flaxseed fibers to a controlled diet will increase excretion of fat and energy, lower blood cholesterol and suppress hunger. Furthermore, a potential difference due to food types will be explored as the flax dietary fiber will be given both as a viscous drink and baked into bread.

## Subjects and methods

### Subjects

Seventeen young subjects (10 women and 7 men) were recruited through advertising at university campuses in the Copenhagen area. The exclusion criteria were as follows: known chronic illnesses (such as diabetes, hypertension, hyperlipidemia, etc.), smoking, exceesive physical activity (> 10 h/week), regular use of medication (oral contraceptives were allowed), use of dietary supplements and food intolerances or dislikes of relevance to the composition of the meals. All study subjects gave written consent after having received verbal and written information about the study. The study was carried out at the Department of Human Nutrition, Faculty of Life Sciences, University of Copenhagen, Denmark, and was approved by the Municipal Ethical Committee of The Capital Region of Denmark in accordance with the Helsinki declaration (KF 01-309595) and registered in the http://clinicaltrials.gov database (NCT00953004).

### Experimental design

The study had a double-blind randomized crossover design, where three different iso-caloric diets were examined during three 7-d dietary intervention periods separated by ≥1 week washout. All food was provided by the Department of Human Nutrition, in daily portions matching the individual subject's energy requirement (ER). Most of the food was pre-portioned. The meals were ready-prepared and only had to be heated up. The subjects were instructed to follow the diet plan very strictly, report any deviation from the diet plan and to maintain their habitual activity level throughout the study. On days 1 and 8 in each intervention period, the subjects came to the Department after an overnight fast (> 10 hours) and abstention from alcohol and physical exercise for 24 hours. Their body weight in underwear was measured to the nearest 0.1 kg (Tanita BWB-600, Japan) and height was assessed at baseline to the nearest 0.5 cm using a wall-mounted stadiometer (Seca, Hultafors, Sweden). Blood pressure measurements were performed on the right arm in the supine position after 10 min of rest with an automatically inflated cuff (UA-787; A & D Co Ltd, Saitama, Japan). Two measurements with at least one minute in between were performed and a mean value was calculated at each visit. Hereafter blood samples were taken. All feces were collected in pre-weighed plastic containers during the last 5 days of each diet period. Transit time was measured by using non-absorbable radio-opaque transit markers. With breakfast on days 1-5 of each diet period, the subjects ingested capsules containing transit markers (20 markers per day), differing in size and shape for each day. Transit time was determined as described elsewhere [20].

#### Diet

A standardized diet was provided for the subjects consisting of breakfast, lunch and evening meals, as well as fruits and snacks. The subjects were allowed to consume water *ad libitum*, and a maximum of two cops of coffee or tea daily. Three daily menus were prepared to vary the diet, but all subjects consumed the same standardized diet on each day. The breakfast meals were served at the Department of Human Nutrition, while the lunch and evening meals were provided for home consumption. On Fridays, the subjects were provided all foods for consumption at home during the weekend. Besides the standardized diet, the subjects were provided with drinks or breads containing a non-fiber thickener (modified corn starch (MCS)) (Thick & Easy ^®^; Fresenius Kabi, Denmark) or flaxseed fiber (Biogin biochemicals co. Ltd, China) three times daily as follows: Control period: MCS drink and plain breads; Flax drink period: Flaxseed fiber drink and plain breads; Flax bread period: MCS drink and Flaxseed fiber breads.

MCS and Flaxseed fibers were provided as powders to be mixed with water and blackberry syrup for immediate consumption 30 minutes prior to all three main meals. The Flaxseed fibers were baked into breads which were consumed as part of the three main meals. No MCS breads were made, as the plain breads were similar in appearance to the Flax breads, whereas the viscosity of Flax drinks needed to be disguised. The dose of MCS was 12 g/10 MJ and the dose of Flaxseed extract was 7.5 g/10 MJ (providing 5.2 g dietary fibers/10 MJ), and were adjusted according to ER.

The energy distribution of the standardized diet including Flax dietary fiber/MCS was as follows: ~15E% from protein, ~29E% from fat, and ~56E% from carbohydrates (Table 1). The energy content of the standardized diet and test supplements were similar in all three periods, and were adjusted to the ER of the individual study subjects. ER was assessed based on assessment of physical activity level (PAL) and DXA scan to measure fat mass (FM) and fat free mass (FFM). ER was calculated as follows, where sex (S) was set to 0 for women and 1 for men:

$$\begin{array}{c} \text{ER}=\text{PAL}\times (0,058\times \text{FFM}(\text{kg})+0,026\times \text{FM}(\text{kg})\;\\ \;-\;0,018\times \text{AGE}(\text{y})+0,615\times \text{S}(0/1)+3,322) \end{array}$$

**Table 1:** The nutrient composition of the three diets, normalized per 10 MJ. ^a^

	Control	Flax drink	Flax bread
Energy (kJ) ^b^	11,040 (10,403)	10,884 (10,092)	11,139 (10,466)
Protein (E%)	14.8	15.1	14.8
Carbohydrates (E%)	56.3	55.6	56.5
Fat (g) ^b^	73.9 (81.5)	73.9 (80.2)	74.1 (81.7)
Fat (E%)	24.8 (29.0)	25.1 (29.4)	24.6 (28.9)
Dietary fiber (g) ^c^	14.1	19.3	19.3

^a ^The nutrient content was estimated using the Dankost 3000 dietary assessment software (Danish Catering Center, Herlev, Denmark). ^b ^The energy and fat contents were measured. The values in parentheses are calculated using Dankost 3000.^c ^Dietary fiber content in the diet was estimated using the Dankost 3000 software, and the dietary fiber content of the flax mucilage was measured.

### Viscosity measurements of drinks

Measurement of viscosity was performed with a StressTech rheometer (Reologica Instruments AB, Sweden) using a cup (26.0 mm) and bob (25.0 mm) geometry over a shear rate range of 1-100 s^-1 ^and temperatures of 10 (temperature at ingestion) and 37°C (physiological temperature). Flax dietary fiber and MCS was mixed with cold water and blackberry syrup until fully suspended and measurements were preformed immediately after preparation of the solutions. Viscosities at 30 s^-1 ^was compared along with the overall viscosity behavior approximated with a flow behavior index *n *from the Power Law model as SS = *c *SR*^n ^*where SS (Pa) is shear stress and SR (s^-1^) is shear rate.

### Appetite registrations and evaluation of gastrointestinal discomfort

To assess appetite sensation, visual analogue scales (VAS) were used. They are 100 mm in length with words anchored at each end, expressing the most positive and the most negative rating, and were used to assess hunger, satiety, fullness, thirst, plus well-being. The subjects were instructed to fill in these questionnaires three times daily before consumption of the drinks (i.e. 30 minutes before each main meal). The use of VAS to assess subjective appetite sensation has however only been validated for use in postprandial single meal studies [21].

At the end of each period, the subjects evaluated gastrointestinal discomfort. They were asked to report whether they had experienced any of the following during the last week: heart burn, reflux, bloating, nausea, stomach pain, stomach rumbling, stomach gas, diarrhea or constipation as well as the severity of these (1 = nothing; 2 = weak; 3 = moderate; 4 = severe; 5 = very severe).

### Analytical procedures

#### Feces

Before analysis, the fecal samples were freeze-dried and homogenized. For each subject, all samples from the same diet period were pooled. Fecal energy was measured by bomb calorimetry (Ika-calorimeter system C4000; Heitersheim, Germany). Total fecal fat was determined using the ANKOM filter bag technique, where fecal samples are acid hydrolyzed with 3 N HCl at 90°C for 1 h, and fat was extracted using petroleum ether (ANKOM^XT15 ^Extraction System, ANKOM Technology, NY, USA).

#### Diet

Samples of the three test diets were homogenized and freeze-dried. The dietary fiber content of the low-fiber diet was estimated using Dankost 3000 dietary assessment software (Dankost 3000, version 2.5, Danish Catering Center, Herlev, Denmark), whereas the dietary fiber content of the flax fiber was measured using AOAC method No. 985.29. Energy and fat content of the three test diets were measured in the same way as described for the fecal samples.

#### Blood samples

Plasma glucose was measured with the use of an enzymatic endpoint method (Hexokinase) (Gluco-quant Glucose/HK, Roche Diagnostics, Basel, Switzerland) using an ABX Pentra 400 chemistry analyzer (ABX Pentra, Horiba ABX, Montpellier, France); intra-assay coefficient of variation (CV) was 1.4%. Concentrations of triacylglycerols and total cholesterol were assessed using colorimetric test kits (Roche TG, Roche Diagnostics GmbH, Mannheim, Germany); intra-assay variations were 0.6% and 0.9%, respectively. HDL-cholesterol was measured using a homogeneous enzymatic colorimetric test kits (Roche HDL-C plus 2^nd ^gene-ration, Roche Diagnostics GmbH, Mannheim, Germany); intra-assay precisions were 1.8%. All analyses were performed on a COBAS MIRA Plus (Roche Diagnostic Systems Inc., Mannheim, Germany). LDL-cholesterol was calculated using the Friedewald equation (LDL-cholesterol = total cholesterol - HDL cholesterol - 0.456 × triacylglycerol) [22]. Insulin was measured by solid-phase, 2-site chemiluminescent immunometric assay (Immulite/immuliter 1000 insulin; Diagnostic Products Corporation, Los Angeles, USA) with the use of an Immulite 1000 analyzer (Diagnostic Products Corporation, Los Angeles, USA). Intra-assay and inter-assay CVs were 2.5% and 4.9%, respectively.

### Statistical analysis

All statistical analyses and calculations were performed using the Statistical Analysis System software package, version 9.1 (SAS Institute inc., Cary, NC, USA). All dependent variables were controlled for homogeneity of variance and normal distribution by investigation of residual plots, normal probability plots and histograms. Analysis of co-variance (ANCOVA) was performed using the PROC MIXED procedure to compare baseline values between the three dietary intervention periods, in which period and diet were modeled as fixed variables; sex and energy intake as covariates and subject was included as a random variable. An ANCOVA analysis was used to investigate the effect of diet, where subject was modeled as a random variable and sex, energy intake, corresponding baseline values and body weight changes were modeled as covariates, and period as well as period × diet interaction were included as fixed variables. A similar model was applied to investigate the effect of diet on body weight, fecal parameters and mean appetite scores, however, without a corresponding baseline value (for fecal parameters and mean appetite scores) and using baseline body weight as a covariate. For all analyses, period × diet interaction was only omitted from the model when p > 0.1. Posthoc pairwise comparisons between diets were made when effect of diet was significant. Results are presented as means ± SD and the statistical significance level defined as p < 0.05.

## Results

Sixteen subjects (seven men and nine women) completed all three diet periods, and one subject (woman) completed only two diet periods and dropped out due to dislike of the diet. The mean age of the subjects was 24.8 ± 3.3 years and mean BMI was 23.8 ± 1.7 kg/m^2^. The subjects' estimated ER ranged from 9 to 17 MJ/d (Table 2). A few subjects reported having omitted food items from the diet plan in the beginning of the first diet period, and a single subject reported having eaten additional food. The individual nutrient intake was adjusted for self-reported deviations from the diet plan using the Dankost 3000. There was no effect of diet on body weight. Blood pressure was in the normal range and was not affected by diet (Table 3).

**Table 2:** Characteristics of the subjects measured at the screening visit prior to inclusion presented as mean ± SD (n = 17)

	Measurement
Age, y	24.8 ± 3.3

Height, m	1.76 ± 0.10
Weight, kg	74.0 ± 11.2
BMI, kg/m^2^	23.8 ± 1.7
Fat free mass, kg	53.9 ± 14.1
Physical activity level (range)	1.65 - 2.11
Estimated energy requirement, MJ/d (range)	9 - 17
Blood pressure, mmHg	
Systolic	120.9 ± 10.9
Diastolic	68.9 ± 6.5

**Table 3:** Mean ± SD of body weight, blood pressure (BP), triglyceride, insulin and glucose concentrations before and after each dietary intervention period and fecal volume, % dry matter (DM), excretion of energy and fat during each dietary intervention period (n = 17 for Control and Flax bread; n = 16 for Flax drink).

	Control	Flax drink	Flax bread	P for diet
Body weight (kg)				NS
Before	71.7 ± 10.1	71.9 ± 10.5	71.7 ± 10.2	
After	71.4 ± 9.9	71.3 ± 10.0	71.1 ± 9.7	
Systolic BP (mmHg)				NS
Before	120.1 ± 9.6	124.1 ± 8.7	122.4 ± 10.2	
After	120.5 ± 9.6	123.9 ± 9.0	123.0 ± 11.1	
Diastolic BP (mmHg)				NS
Before	68.4 ± 5.9	70.1 ± 5.9	69.1 ± 8.1	
After	67.8 ± 5.2	69.5 ± 6.8	69.0 ± 7.0	
Triglyceride (mmol/l)				NS
Before	0.85 ± 0.31	0.87 ± 0.35	0.76 ± 0.25	
After	0.71 ± 0.20	0.75 ± 0.22	0.71 ± 0.24	
Insulin (pmol/l)				NS
Before	26.2 ± 10.7	27.4 ± 10.2	26.3 ± +11.8	
After	28.9 ± 10.4	26.2 ± 10.6	28.2 ± 12.8	
Glucose (mmol/l)				NS
Before	5.3 ± 0.3	5.4 ± 0.3	5.3 ± 0.4	
After	5.3 ± 0.2	5.3 ± 0.3	5.3 ± 0.3	

**Fecal parameters**				
Transit time (h)	49.7 ± 3.1	43.9 ± 3.3	47.9 ± 3.3	NS
Fecal excretion (g/d)	121 ± 12	142 ± 13	119 ± 13	NS
Excretion of DM (g/d)	28 ± 2.2	33 ± 2.3	30 ± 2.3	NS
% DM in feces	24.9 ± 1.1	25.0 ± 1.1	26.9 ± 1.1	NS
Energy excretion (kJ/d)	565 ± 45 ^a^	694 ± 45 ^b^	606 ± 44 ^ab^	0.053
% Energy excreted	4.51 ± 0.38 ^a^	5.51 ± 0.39 ^b^	4.92 ± 0.39 ^ab^	0.061
Fat excreted (g/d)	3.20 ± 0.30 ^a^	4.96 ± 0.31 ^b^	3.76 ± 0.31 ^a^	< 0.001
% Fat excreted	3.46 ± 0.33 ^a^	5.21 ± 0.33 ^c^	4.09 ± 0.34 ^b^	< 0.001

^a, b, c ^Different letters in same row indicate significantly different values; p < 0.05.

### Viscosity of the samples

The viscosity of the reference MCS and Flax drink was 6 mPas and 528 mPas, respectively, at the moment of ingestion (10°C). With a flow behavior index of *n*≈1 the MSC drink was Newtonian whereas the flax drink was shear thinning (*n *= 3). At physiological temperature (37°C) the viscosity of the flax drink dropped to 361 mPas, whereas the MSC drink remained at same level. Both solutions exhibited same flow behavior as for 10°C.

### Blood measurements

**Figure 1:**
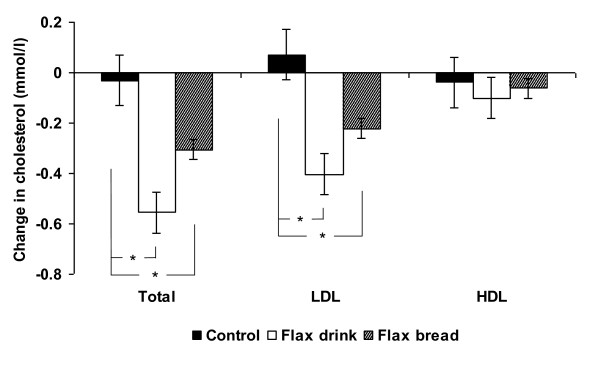
**Change in total, LDL and HDL cholesterol after 7 days consumption of a control diet, a Flax drink diet and a Flax bread diet**. Total and LDL cholesterol was significantly reduced after the Flax drink and Flax bread diets compared to control (p < 0.05) (n = 17 for Control and Flax bread; n = 16 for Flax drink).

Flax drink lowered fasting total-cholesterol and LDL-cholesterol by 12 and 15%, respectively, compared to Control (p < 0.01), whereas smaller declines were observed for Flax bread in comparison to Control (7 and 9%, p < 0.05) (Figure 1). No effect was seen on fasting triacylglycerol, HDL-cholesterol, glucose or insulin (Table 3).

### Fecal parameters

Daily fecal volume and excretion of DM did not differ between diets. However, fecal excretion of energy was affected by diet (p < 0.05). Post-hoc pairwise comparison showed that a 23% (~129 kJ/d) greater fecal excretion of energy was seen with consumption of Flax drink compared to the Control (p = 0.05), and the proportion of energy excreted of intake increased correspondingly (p < 0.05). Also, fecal fat excretion was affected by diet (p < 0.01) as 4.96 ± 0.31 g fat/d was excreted when consuming the Flax drink diet as compared to only 3.20 ± 0.33 g fat/d with the Control diet, corresponding to a 55% increase. The percentage of fat intake excreted increased correspondingly (p < 0.01). No significant effect of Flax bread was observed on any of the fecal parameters compared to the Control diet. Transit time was not significantly affected by diet.

### Subjective appetite sensation and reporting of gastrointestinal discomfort

**Figure 2:**
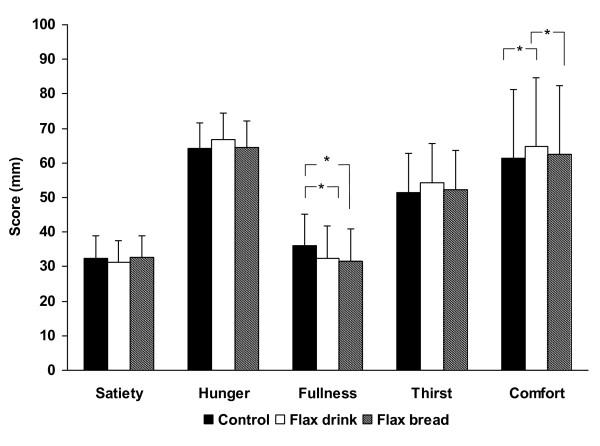
**Mean ratings of sensation of satiety, hunger, fullness, thirst and comfort during days 2-7 of consuming a control diet, a Flax drink diet and a Flax bread diet**. Sensation of fullness was rated greatest with the Control diet (p < 0.05) and comfort was rated greatest with the Flax drink diet (p < 0.05) (n = 17 for Control and Flax bread; n = 16 for Flax drink).

There was no difference in the appetite ratings between days, thus data from day 2-7 were pooled (because day 1 ratings deviated from all other days) and are presented as mean ratings (Figure 2). Overall, ratings of satiety, hunger, fullness, thirst and comfort did not differ between dietary intervention apart from ratings of fullness being higher throughout the Control period compared to both Flax drink and Flax bread (p < 0.01). This may be linked to the slightly higher energy intake during the Control period (Table 1). During the Control period, the subjects consumed a drink containing MCS, a digestible polysaccharide modified in order to induce viscosity. This product did, however induce increased bloating in some subjects, which may have interfered with their perception of fullness. Thus, we conducted a subset analysis after exclusion of subjects reporting increased bloating during any dietary intervention period, which eliminated the differences in fullness ratings (data not shown). Also, a difference between dietary interventions on comfort ratings was observed (p = 0.03), where subjects had higher comfort ratings when consuming the Flax drink compared to Control (p < 0.05). The most frequent gastrointestinal discomforts reported were bloating, stomach rumbling and gas, and for most of the reported discomforts, no differences between diets were observed when looking at the sum of severity of ratings, whereas gastrointestinal discomfort overall appeared more often during the Control period and Flax drink period. Bloating occurred more severely and frequently in the Control period, and stomach pain and rumbling occurred more frequently during the Flax drink period (Table 4).

**Table 4:** Mean ratings of gastrointestinal side effects for each dietary intervention (Control, Flax drink or Flax bread) recorded at the end of each period (n = 17 for Control and Flax bread; n = 16 for Flax drink).

Number of study subjects reporting side effects										
	**Sum**	**Heartburn**	**Reflux**	**Bloating**	**Nausea**	**Pain**	**Rumbling**	**Gas**	**Diarrhea**	**Constipation**
Control	32	3	2	6	4	1	3	6	3	4
Flax drink	34	3	2	3	3	4	6	6	3	4
Flax bread	22	4	3	1	1	1	1	5	2	4
										
**Mean degree of symptom (1 = nothing; 5 = very severe)**										

	Sum	Heartburn	Reflux	Bloating	Nausea	Pain	Rumbling	Gas	Diarrhea	Constipation
Control	13.1	1.7	1.3	1.8	1.5	1.1	1.5	1.5	1.3	1.5
Flax drink	12.2	1.4	1.1	1.4	1.2	1.4	1.5	1.6	1.4	1.2
Flax bread	12.0	1.7	1.4	1.1	1.3	1.1	1.2	1.6	1.3	1.4

## Discussion

The results of the present study demonstrate that consumption of 5 g of dietary fibers from flaxseeds daily for one week significantly increased fecal excretion of fat and reduced total and LDL-cholesterol markedly. Despite similar doses of dietary fiber, the effect of the dietary fiber was less pronounced when incorporated into bread than when administered as a drink, which emphasizes the importance of food matrix.

We observed a lowering of both total-cholesterol and LDL-cholesterol by 12 and 15%, respectively, within just seven days in young healthy adults with normal blood cholesterol concentrations. It can be speculated that the effect may be overestimated as the short intervention period does not allow for equilibrium to occur, but the short duration was chosen due to the high level of controlled diet and the fact that fecal parameters were the main end point. However, in support of our findings, effects of similar magnitude are reported in a recently published study, in which 5 g of flaxseed gum per day for three months reduced total and LDL cholesterol by 10 and 16%, respectively in type 2 diabetics [8], although one could expect a more pronounced effect among diabetics with dyslipidemia. A recent meta-analysis on the effects of flaxseed on blood lipids showed that flaxseed consumption lower both total and LDL-cholesterol, whereas flaxseed oil does not, and the role of lignans is still controversial [23]. Thus, the responsible component for the assumed cardioprotective effect of flaxseeds may well be the fiber component. The 5 g dose of fibers used was relatively low compared to other studies. Flaxseeds contain ~30% dietary fibers, with one third being viscous; thus the dose used would correspond to ~50 g of whole flaxseeds or slightly less, as non-viscous fibers may also contribute. The most plausible mechanism of action is through an interference with bile acid metabolism, where an increased intraluminal viscosity can 1) hinder micelle formation and thus diminish lipid uptake and 2) inhibit re-uptake of bile acids causing increased hepatic synthesis of bile acids which diverts cholesterol away from lipoprotein synthesis in the liver, thereby reducing serum cholesterol [7]. Short chain fatty acid production has also been proposed to play a role, and although flaxseed dietary fibers have been shown to be highly fermentable in rats [24], this mechanism has not been confirmed in humans [7]. Flax drink at the moment of ingestion (10°C) and at physiological temperature (37°C) showed significant higher viscosity (300-500 fold) compared to control MCS drink. Although it is possible that the flax dietary fibers may be subject to depolymerization and hence loss of viscosity with ingestion, we assume that the overall luminal viscosity is higher for flax dietary fibers compared to control (MCS) drink.

Fecal energy excretion increased by 129 kJ/d with Flax drink consumption compared to Control. The amount of fat and energy which escapes digestion may appear small in numbers, but corresponds to ~47 MJ excreted per year and thus of relevance in the prevention of weight gain as a decrease in energy uptake of this magnitude equals a difference in body weight of ~1.6 kg. The increase in fecal energy and fat excretion is in accordance with our previous studies on flaxseed fibers showing a reduction in energy digestibility and weight gain in growing Wistar rats fed a diet with 10% of flaxseed dietary fibers (Kristensen M, Knudsen KEB, Jørgensen H, Oomah D, Bügel S, Toubro S, Tetens I, Astrup A. Flaxseed dietary fibers reduce apparent energy and fat digestibility and weight gain in growing rats, submitted). Studies using other viscous dietary fibers have shown similar results [25-27], and Eastwood and colleagues (1986) compared different dietary fiber sources and found that gum tragacanth, gum arabic and raw carrot increased faecal fat excretion, whereas non-viscous potato dietary fibers did not [28]. In a study on broiler chickens fed either flaxseeds or demucilaged flaxseeds found that removing the mucilage layer reduced energy utilization and viscosity of the jejunal digesta [15], strongly suggesting that the physiological effects are closely linked to their ability to form viscous gels upon hydration. In contrast, a recent study found that insoluble (cereal) rather than soluble fiber (guar gum) increased fecal energy excretion in growing mice [29]. Interestingly, the soluble fiber also resulted in a larger weight gain than insoluble fibers, which the authors linked to fermentation of soluble fibers leading to increased energy extraction. This cannot be extrapolated to humans due to differences between species, but does warrant further research into the importance of large intestinal contributions to energy balance.

Appetite sensation was assessed three times daily throughout the present study, and there was an overall trend towards increased fullness during the Control period compared to both Flax drink and bread. This was unexpected as we have previously observed that flax fibers significantly increases both satiety and fullness [19] and no studies on viscous dietary fibers indicate that they should induce hunger. However, we believe that the Control drink based on MCS may have caused bloating, which may have been mistaken for fullness. This is supported by the subset analysis on subjects not reporting increased bloating during any dietary intervention period, which eliminated the differences in fullness ratings. Appetite sensation was assessed 30 minutes prior to main meals; however, the subjects were not instructed to have their lunch and dinner at fixed time points, which may have influenced the results.

One of the objectives of the present study was to explore the effect of food matrix on physiological responses. We found that the effect of Flax bread was less pronounced than with a similar dose of dietary fiber provided as a drink, both with regard to cholesterol-lowering properties and reduction of apparent fat digestibility, although only the latter was significantly different between Flax drink and Flax bread. An effect of food matrix was also seen in a study, in which β-glucan enriched juices produced a more pronounced effect on total and LDL-cholesterol compared to β-glucan enriched cookies [30], but in general evidence on the importance of food matrix are lacking. The different effects observed between drinks and baked products may be due to differences in their ability to induce viscosity resulting from either reduced hydration of the dietary fiber or reduced molecular weight in the baked products due to processing and/or storage, as reported for β-glucans [31]. Further, Flax fiber's ability to directly adsorb fat and bile acids may have been compromised by processing or the inclusion of a viscous drink with MCS in the Flax bread period may have interfered with the water-interaction of the flaxseed fibers.

In conclusion, we studied the effect of flax fiber-enriched drinks and breads on fecal energy and fat excretion, blood lipids as well as subjective appetite sensation. We found that addition of a flax dietary fiber extract rich in viscous dietary fibers significantly increased fat excretion and lowered total and LDL-cholesterol although no effect on appetite was observed. Viscous flaxseed fibers appear useful for lowering blood cholesterol and may play a role in energy balance; however, food type and/or processing may be of importance.

## Competing interests

The University of Copenhagen has a patent pending concerning the present research and Arne Astrup is a consultant for Basic Research, Salt Lake City, Utah, USA. All other authors have no competing interests.

## Authors' contributions

MK participated in design of the study, performed the statistical analyses and drafted the manuscript. MGJ participated in design of the study. JA, KENP and LS performed the experimental work, MSM performed the viscosity measurements. AA participated in design of the study All read and approved the final manuscript.
